# Rapid model exploration for complex hierarchical data: application to pharmacokinetics of insulin aspart

**DOI:** 10.1002/sim.6536

**Published:** 2015-05-26

**Authors:** Robert J. B. Goudie, Roman Hovorka, Helen R. Murphy, David Lunn

**Affiliations:** ^1^ MRC Biostatistics UnitCambridge Institute of Public HealthCambridgeU.K; ^2^ Department of PaediatricsUniversity of CambridgeCambridgeU.K; ^3^ Wellcome Trust‐MRC Institute of Metabolic Science, Level 4 Metabolic Research LaboratoriesUniversity of CambridgeCambridgeU.K; ^4^ NIHR Cambridge Biomedical Research CentreCambridgeU.K

**Keywords:** Bayesian hierarchical models, variable selection, Markov chain Monte Carlo, pharmacokinetics, insulin

## Abstract

We consider situations, which are common in medical statistics, where we have a number of sets of response data, from different individuals, say, potentially under different conditions. A parametric model is defined for each set of data, giving rise to a set of random effects. Our goal here is to efficiently explore a range of possible ‘population’ models for the random effects, to select the most appropriate model. The range of possible models is potentially vast, because the random effects may depend on observed covariates, and there may be multiple credible ways of partitioning their variability. Here, we consider pharmacokinetic (PK) data on insulin aspart, a fast acting insulin analogue used in the treatment of diabetes. PK models are typically nonlinear (in their parameters), often complex and sometimes only available as a set of differential equations, with no closed‐form solution. Fitting such a model for just a single individual can be a challenging task. Fitting a joint model for all individuals can be even harder, even without the complication of an overarching model selection objective. We describe a two‐stage approach that decouples the population model for the random effects from the PK model applied to the response data but nevertheless fits the full, joint, hierarchical model, accounting fully for uncertainty. This allows us to repeatedly reuse results from a single analysis of the response data to explore various population models for the random effects. This greatly expedites not only model exploration but also cross‐validation for the purposes of model criticism. © 2015 The Authors. Statistics in Medicine published by John Wiley & Sons Ltd.

## 1 Introduction

Consider a hierarchical data set, where we have a number of sets of response data, from different patients perhaps. We wish to apply a parametric model to each individual's data set and then define a ‘population’ model relating all of the individual‐level parameters (random effects) together. There may be a variety of credible models for the random effects, and it is important to fully explore a range of possibilities. For example, the parameters may depend on observed covariates, or there may be different ways of partitioning their variability across levels of the hierarchy. However, such exploration can be cumbersome and time‐consuming, especially when individual‐level models are complex. For example, pharmacokinetic models are typically nonlinear (in their parameters), often have complex functional forms and are sometimes only available as a set of differential equations, with no closed‐form solution. Fitting such a model for just a single individual can be a challenging task. Fitting a joint model for all individuals can be even harder, even without the complication of an overarching model selection objective.

We aim in this paper to develop a methodology for efficiently fitting a range of population models (including covariate selection) to the parameters (random effects) from individual‐level models. We describe a *two‐stage* approach that decouples inference on the population model from inference on each individual‐level model but nevertheless fits the full, joint, hierarchical model, accounting fully for uncertainty. In the first stage, we estimate independent posterior distributions for the parameters in each individual‐level model using Markov chain Monte Carlo (MCMC). We then store the resulting samples for use in the second stage, where they form ‘proposal distributions’ for the individual‐level parameters in the full hierarchical model. The parameters are then updated via Metropolis–Hastings steps with an acceptance probability that is independent of the individual‐level likelihood. This means that stage 2 is very efficient, allowing a wide range of models to be easily explored. In particular, our approach facilitates rapid exploration of covariate models using *reversible jump* MCMC, as well as exploration of different models for the variance components. It also facilitates criticism of the various population models via cross‐validation techniques. Our approach is suited to situations in which the number of observations for each individual exceeds the number of parameters in the individual‐level model and so is most likely to be useful in early clinical studies, in which detailed individual‐level data are available.

We use this methodology to study four parameters relating to insulin kinetics in pregnant women with type 1 diabetes, using data from two clinical studies. We consider covariate selection models that enable identification of covariates that may be related to the kinetic parameters, as well as different structures for the hierarchical model, up to four levels. We believe that the complexity of our analysis would render it impracticable without the proposed two‐stage methodology. Our two‐stage approach is implemented in extensions to the OpenBUGS software 1, 2, which is freely available from www.openbugs.net.

Rapid‐acting insulin analogues (such as insulin aspart and insulin lispro) can assist in safely optimising glucose control 3, 4, 5, but little is known about their pharmacokinetics and reproducibility in pregnancy. A significant gestational delay of approximately 30min in time‐to‐peak plasma aspart concentration from early to late pregnancy has been previously described 6. The aims of the study reported herein were to explore the relationship between aspart pharmacokinetics and clinical/demographic factors for subjects with type 1 diabetes undergoing continuous subcutaneous insulin infusion (CSII) during pregnancy and to assess reproducibility within and between subjects.

Basic clinical results from some of these analyses have been reported previously 7. Here, we present and explore more thoroughly the statistical and methodological issues.

## 2 Data

Our data are from two 24‐h trials of insulin aspart for pregnant women with type 1 diabetes conducted between March 2009 and April 2011 8, 9. Study protocols were approved by the Research Ethics Committee, and all participants provided written informed consent.

During each study, women arrived at the clinical research facility (Addenbrooke's Hospital, Cambridge, UK) at midday and were monitored for 5h after dinner on day 1 and breakfast on day 2. In study 1, 10 women were studied on two occasions: in early (12–16weeks) and in later (28–32 weeks) gestation 8, with standardised dinner and breakfast, and under sedentary conditions. In study 2, 12 women were studied on two occasions in mid gestation (12–33weeks) with standardised meals, snacks and exercise (3 × 20‐min walks at 14:00, 19:30 and 09:00h; and 2 × 50‐min treadmill sessions at 15:00 and 09:30h) 9.

A CSII delivering aspart maintained stable fasting and pre‐meal glycaemic conditions during each visit using either closed‐loop or conventional CSII. Under closed‐loop CSII, the basal rate was adjusted every 15min using continuous glucose measurements, whereas with conventional CSII, the women set temporary basal rates and used correction boluses according to capillary glucose measurements. The basal infusion rate was recorded from 14:00h on day 1 in the first study and from 00:00h on day 1, before the study started, in study 2. The median (interquartile range) infusion rate was 0.6(0.1–1.2) international units (U) per hour. All prandial insulin boluses were calculated according to capillary glucose levels and were initiated at 18:00h on day 1 and 11:00h on day 2. The median (interquartile range) prandial bolus dose was 8.9(6.5–12)U. We assume boluses infused steadily over a 1‐min period, apart from eight boluses, which were delivered over longer periods. Insulin concentration readings were recorded from 16:30h on day 1 in the first study and from 14:00h on day 1 in the second study. Plasma insulin concentration was measured every 10min for 90min post‐meal, every 15min for 1.5–5h post‐meal and at 15‐ to 30‐min intervals at other times, providing an average of 59 measurements per woman. Plasma insulin concentration was measured by an immunochemiluminometric assay (Invitron, Monmouth, UK; intra‐assay coefficient of variation (CV) 4.7% and inter‐assay CV 7.2–8.1%). Further details of study procedures are reported elsewhere 8, 9.

We consider each mealtime separately so that the basic unit of study is a time series of insulin concentrations (a ‘profile’) over a period around the evening (17:30–23:00h on day 1) or morning (06:30–12:00h on day 2) meal from a particular visit of a pregnant woman. A total of 22 women underwent two visits, each involving two meals; this gave rise to 22 × 2×2 = 88 profiles to model.

Thirteen clinical and demographic factors were examined. These factors, which were considered time invariant at the profile‐level, are summarised in Table 1.

**Table 1:** Summary of the 13 clinical and demographic factors.

Factor	Summary
Maternal age (years)	32(4.5)
Body mass index (kg/m^2^)	27(3.3)
Glycated haemoglobin (HbA1c) at booking (%)	7.1(1.0)
Duration of diabetes (years)	18(8.6)
Pregnancy gestation (weeks)	22(6.5)
Expected total daily dose (units/day)	55(18)
Peak bolus rate (units/hour)	9.3(5.0)
Recruited at Kings College [baseline Addenbrookes] (indicator)	5/22 women
Bolus delivered over longer than 1min (indicator)	8/88 profiles
Multiple boluses (indicator)	17/88 profiles
Closed‐loop basal insulin delivery (indicator)	24/88 profiles
Study 1 [baseline study 2] (indicator)	10/22 women
Breakfast [baseline dinner] (indicator)	44/88 profiles

Means (standard deviation) shown for continuous factors, and the number of profiles/women (out of total number) with that attribute are shown for dichotomous indicators.

## 3 Methods

### 3.1 Profile‐level model

#### 3.1.1 Mechanistic model

**Figure 1:**
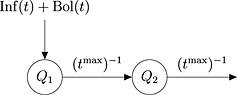
Two‐compartment model for insulin kinetics, in which *Q*_1_(*t*) and *Q*_2_(*t*) represent the insulin masses in two subcutaneous tissue compartments, representing the delay in absorption into the blood. Inf(*t*) and Bol(*t*) represent insulin input via continuous infusion and supplemental boluses, respectively.

We use a two‐compartment model to represent insulin kinetics, as shown in Figure 1. At time *t*, *Q*_1_(*t*) and *Q*_2_(*t*) represent the insulin masses (in international units, U) in two subcutaneous tissue compartments, representing the delay in absorption into the blood. A controlled infusion of insulin enters compartment 1 at a rate Inf(*t*) (mU/min), supplemented by boluses of insulin, which we model as entering at a rate Bol(*t*) (mU/min). Insulin leaves both compartments according to a first‐order process, with rate constant (*t* ^max^)^−1^, where *t* ^max^ is the time‐to‐peak insulin concentration in minutes. This gives rise to a pair of ordinary differential equations:

(1) $$\frac{dQ_{1}(t)}{\mathrm{dt}}=\mathrm{Inf}(t)+\text{Bol}(t)-Q_{1}(t)/t^{\max},$$

(2) $$\frac{dQ_{2}(t)}{\mathrm{dt}}=\frac{Q_{1}(t)-Q_{2}(t)}{t^{\max}},$$

with *Q*_1_(0) = *Q*_2_(0) = 0, where *t* = 0 corresponds to midnight on day 1 before the trial starts. The run‐in time between *t* = 0 and the time *t* = *t* ^start^, when the trial starts, allows the states *Q*_1_(*t* ^start^) and *Q*_2_(*t* ^start^) to become independent of *Q*_1_(0) and *Q*_2_(0). Equations (1)–(2) do not, in general, have a closed‐form solution. In this paper, we obtain a numerical solution, instead, via the Cash–Karp Runge–Kutta method 10.

We base our regression function on the observable quantity in these trials, which is plasma insulin concentration. This is assumed to equilibrate instantaneously with the efflux from compartment 2 and is given by *Q*_2_(*t*)/(*t* ^max^×*w* *t* × MCR), where *wt* denotes the patient's body weight (kilogramme) and MCR is the *metabolic clearance rate* per unit of body weight: MCR is the volume of plasma (l) cleared of insulin per minute, per kilogramme. To account for any long‐acting insulin taken before the start of the study (which continues to have an effect for around 24h), we add a linear, ‘residual insulin’ term to our regression function. We assume that residual insulin concentration changes at a rate *a* (pmol/l/min) and that the post‐prandial concentration at time *t* ^end^ (5h post‐meal) is *b* (pmol/l). The regression function is then given by

$$\mu (\theta ,z,t)=\frac{Q_{2}(t)}{t^{\max}\times \mathrm{wt}\times \text{MCR}}+a\left(t-t^{\text{end}}\right)+b,$$

with unknown parameters *θ* = (*t* ^max^,MCR,*a*,*b*)′ and observed data *z* = (*w* *t*,*t* ^end^)′.

#### 3.1.2 Observation model

Denote by *y*_*ijkm*_ the *m*th measured plasma insulin concentration, taken at time *t*_*ijkm*_, for individual *i* during and the following meal *k* of visit *j* (*i* = 1,…,*N*, *j* = 1,…,*J*_*i*_, *k* = 1,…,*K*_*ij*_, *m* = 1,…,*T*_*ijk*_). We assume

$$y_{\mathrm{ijkm}}∼\text{N}\left(\mu_{\mathrm{ijkm}},\sigma_{\mathrm{ijkm}}^{2}\right),\;\;\;\mu_{\mathrm{ijkm}}=\mu \left(\theta_{\mathrm{ijk}},z_{\mathrm{ijk}},t_{\mathrm{ijkm}}\right),$$

where *θ*_*ijk*_ and *z*_*ijk*_ are *profile‐specific* vectors of unknown parameters and data, respectively. In addition, the residual variance is given by

$$\sigma_{\mathrm{ijkm}}^{2}=\kappa_{\mathrm{ijk}}^{2}+\lambda_{\mathrm{ijk}}^{2}\times \mu_{\mathrm{ijkm}}^{2},$$

which combines additive and multiplicative variance models, allowing for increases in measurement error above some baseline level as the modelled insulin concentration increases.

### 3.2 Population model

We wish to make inferences about the unknown parameters *t* ^max^, MCR, *a* and *b*. In particular, we are interested in establishing whether any relationships exist between the parameters and the observed covariates, and in quantifying their variabilities both within and between women. We begin by making separate distributional assumptions for each component (indexed by *l* = 1,…,4) of the parameter vector *θ*_*ijk*_:

$$\theta_{\mathrm{ijkl}}∼\mathrm{LN}\left(\eta_{\mathrm{ijkl}},\sigma_{\theta_{l}}^{2}\right),\;l=1,2,\;\;\;\theta_{\mathrm{ijkl}}∼\text{N}\left(\eta_{\mathrm{ijkl}},\sigma_{\theta_{l}}^{2}\right),\;l=3,4,$$

where LN(.,.) denotes a *log‐normal* distribution with first and second parameters corresponding to mean and variance, respectively, on the log‐scale. Note that we specifically avoid making a *multivariate* assumption for the whole *θ*_*ijk*_ vector, for reasons that we will discuss later (Section 5). By choosing different forms for *η*_*ijkl*_, we can specify a variety of population models for the *θ*_*ijkl*_s. In particular, we consider one‐level, two‐level and three‐level population models as follows. In each case, we consider models both with and without covariates included.

#### 3.2.1 One‐level models

Here, we assume that the profile‐specific parameters *θ*_*ijkl*_ are all conditionally independent, given a set of *global* parameters (or fixed effects). Hence

$$\eta_{\mathrm{ijkl}}=\left\{\begin{array}{ll} \varphi_{l}+W_{\mathrm{ijkl}}\beta_{l} & \;\text{with covariates}\\ \varphi_{l} & \;\text{without} \end{array}\right.$$

where*W*_*ijkl*_is a row‐vector containing observed values (for individual *i* at meal *k* of visit *j*) of the covariates chosen as predictors for the *l*th kinetic parameter (*l* = 1,…,4); we discuss this further in Section 3.2.4. In this model, *φ*_*l*_ represents the global mean (or intercept if covariates are included in the model), and $\sigma_{\theta_{l}}^{2}$ represents the *total* variability (after any controlling for covariates) of parameter (or log‐parameter) *l*.

#### 3.2.2 Two‐level models

Here, we acknowledge that profiles from the same woman may be correlated. It is tempting to achieve this by introducing patient‐specific intercept and gradient parameters. However, with only four profiles from each woman, it is not very realistic to attempt to estimate patient‐specific covariate effects, and so, we allow only the intercept to vary between women:

$$\eta_{\mathrm{ijkl}}=\left\{\begin{array}{ll} \psi_{\mathrm{il}}+W_{\mathrm{ijkl}}\beta_{l} & \;\text{with covariates}\\ \psi_{\mathrm{il}} & \;\text{without} \end{array}\right.$$

with $\psi_{\mathrm{il}}∼\text{N}(\varphi_{l},\sigma_{\psi_{l}}^{2})$, *i* = 1,…,*N*. Here, $\sigma_{\psi_{l}}^{2}$ measures the between‐patient variability (for parameter *l*), and $\sigma_{\theta_{l}}^{2}$ now represents the within‐patient variability.

#### 3.2.3 Three‐level models

Additionally, we might acknowledge that profiles from the same visit for a given woman may be correlated. Alternatively, profiles corresponding to the same mealtime for a given woman may be correlated. We might, therefore, allow visit‐specific or mealtime‐specific intercepts via

$$\eta_{\mathrm{ijkl}}=\left\{\begin{array}{ll} \chi_{\mathrm{ijl}}^{V}+W_{\mathrm{ijkl}}\beta_{l} & \text{with covariates}\\ \chi_{\mathrm{ijl}}^{V} & \text{without} \end{array}\right.\;\;\;\text{or}\;\;\;\eta_{\mathrm{ijkl}}=\left\{\begin{array}{ll} \chi_{\mathrm{ikl}}^{M}+W_{\mathrm{ijkl}}\beta_{l} & \text{with covariates}\\ \chi_{\mathrm{ikl}}^{M} & \text{without} \end{array}\right.,$$

respectively. Here, $\chi_{\mathrm{ijl}}^{V}∼\text{N}\left(\psi_{\mathrm{il}},\sigma_{\chi_{l}^{V}}^{2}\right)$ or $\chi_{\mathrm{ikl}}^{M}∼\text{N}\left(\psi_{\mathrm{il}},\sigma_{\chi_{l}^{M}}^{2}\right)$, and again $\psi_{\mathrm{il}}∼\text{N}\left(\varphi_{l},\sigma_{\psi_{l}}^{2}\right)$, *i* = 1,…,*N*. In both cases, *ψ*_*il*_ represents the patient‐specific mean intercept for parameter *l*, and $\sigma_{\psi_{l}}^{2}$ measures the between‐patient variability. The terms $\sigma_{\chi_{l}^{V}}^{2}$ and $\sigma_{\chi_{l}^{M}}^{2}$, respectively, measure the variability between visits and between mealtimes (for a given individual) of the random intercepts for parameter *l*. In the case of visit‐specific intercepts, $\sigma_{\theta_{l}}^{2}$ now measures the within‐visit variability, whereas in the case of mealtime‐specific intercepts, $\sigma_{\theta_{l}}^{2}$ measures the within‐mealtime variability.

**Figure 2:**
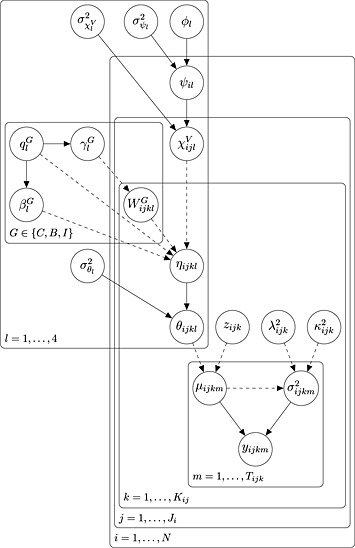
Directed acyclic graph representation of the three‐level (visit) model with covariates. Each variable in the model is represented by a node, and links between the nodes denote direct dependence. Stochastic and deterministic (logical) dependence are represented by solid and dashed lines, respectively. Variables that are repeated are enclosed by ‘plates’ with the plate label denoting the range of repetition (e.g. *i* = 1,…,*N*).

Figure 2 shows a graphical model representation of our statistical model in the case of three‐level (visit) model with covariates.

#### 3.2.4 Covariate selection

Suppose we have *c* available covariates in total and we arrange their observed values in an *n* × *c* matrix *X*, where *n* is the total number of profiles. We expect the importance of each covariate may differ between parameters of the mechanistic model, and thus, *η*_*ijkl*_ for each *l* = 1,…,4 may be a function of a different subset of the predictors. We represent the selected subset of covariates by a vector $\gamma_{l}={\left(\gamma_{l1},\ldots ,\gamma_{lq_{l}}\right)}^{\prime }$. This gives the column indices in *X* of the selected covariates. Let *W*_*l*_ denote the corresponding *n* × *q*_*l*_ design matrix and *W*_*ijkl*_ denote the row of *W*_*l*_ corresponding to meal *k* at visit *j* for individual *i*. Thus, the appropriate linear predictor term for inclusion in *η*_*ijkl*_ is *W*_*ijkl*_ *β*_*l*_, where *β*_*l*_ is a vector of *q*_*l*_ regression coefficients. In this paper, we treat each *q*_*l*_, *γ*_*l*_ and *β*_*l*_ as unknown parameters and estimate their values using *reversible jump* MCMC 11, 12. Note that we standardise (and centre) the continuous covariates in *X* so that they are assessed in covariate selection on the same scale.

*Interactions.* Preliminary exploratory analyses suggested that parameters may differ between study and/or mealtime. However, when these covariates were included in *X*, neither was selected with high probability. We therefore decided to explore possible interactions. We allow for this by replacing study and mealtime in *X* with indicator variables for the following four combinations: study 1 breakfast, study 1 dinner, study 2 breakfast and study 2 dinner. We then choose a suitably weighted prior for the number of these indicators allowed in the model simultaneously, as discussed in Section 3.3.2.

### 3.3 Priors

#### 3.3.1 Variance components

The parameters of the residual variance model are treated as nuisance parameters and are assigned the following vague priors:

(3) $$\kappa_{\mathrm{ijk}}∼\text{U}(0,100),\;\;\;\lambda_{\mathrm{ijk}}∼\text{U}(0,1),\;\;\;i=1,\ldots ,N,\;j=1,\ldots ,J_{i},\;k=1,\ldots ,K_{\mathrm{ij}}.$$

The upper bound of 1 for the *λ*_*ijk*_s is chosen as residuals larger in magnitude than the modelled concentration are implausible in this setting. The remaining variability parameters are all assigned vague uniform priors on the standard deviation scale:

$$\sigma_{\theta_{l}},\sigma_{\psi_{l}},\sigma_{\chi_{l}^{V}},\sigma_{\chi_{l}^{M}}∼\text{U}(0,100),\;\;\;l=1,\ldots ,4.$$

#### 3.3.2 Fixed effects

The global intercepts (or means if no covariates are included in the model), *φ*_*l*_, *l* = 1,…,4, are assigned vague normal priors with zero mean. The variance for the log‐transformed parameters (*l* = 1,2) is 100^2^, whereas 1000^2^ is chosen for *l* = 3,4. The following prior distributions are assumed for the regression coefficients:

$$\beta_{\mathrm{lv}}∼\text{N}\left(0,\sigma_{\beta_{\mathrm{lv}}}^{2}\right),\;\;\;l=1,\ldots ,4,\;v=1,\ldots ,q_{l}.$$

However, in reversible jump MCMC, the elements of each *β*_*l*_ will play different roles in the covariate model from one iteration to the next; that is, a given element may correspond to various different covariates during evolution of the simulated Markov chain. Hence, it seems appropriate to choose the same prior variance for each element. In light of this, we can standardise the covariates so that the model treats them all equally. But we may still wish to allow for more or less diffuse priors for different types (or groups) of covariate. We thus decompose the linear predictor into separate terms for the continuous, binary and ‘study–mealtime‐interaction’ covariates:

$$W_{\mathrm{ijkl}}\beta_{l}=W_{\mathrm{ijkl}}^{C}\beta_{l}^{C}+W_{\mathrm{ijkl}}^{B}\beta_{l}^{B}+W_{\mathrm{ijkl}}^{I}\beta_{l}^{I},$$

where the *C*, *B* and *I* superscripts denote subvectors of *W*_*ijkl*_ and *β*_*l*_, corresponding to continuous, binary and interaction covariates, respectively, and the same prior variance is specified for all elements of each subvector of *β*_*l*_.

The prior standard deviation for coefficients corresponding to each covariate group is given by Δ*β*_*l*_/1.96Δ*x*, where Δ*β*_*l*_ is the width of the range of plausible values for the *l*th kinetic parameter (or log‐parameter) and Δ*x* is the width of the range of values for that covariate‐type: Δ*x* = 1 for binary covariates (including study–mealtime interactions) and Δ*x*≈2 × 1.96 for standardised continuous covariates. This is based on the assumption that the minimum and maximum plausible gradients define a 95% prior interval. Note that, lacking other prior information, we set Δ*β*_*l*_ to the range of the corresponding stage 1 posterior medians, and so, there is an element of using the data twice. While undesirable, this is necessary because the chosen prior variance influences the probability of inclusion of covariates in the model; hence, an informative prior is essential here.

The parameters associated with each covariate group $\left(q_{l}^{G}=\dim\left(\overset{G}{\underset{l}{\beta }}\right),\overset{G}{\underset{l}{\gamma }},\overset{G}{\underset{l}{\beta }},\;\text{with}\;G\in \{C,B,I\}\right)$ are updated in the overall MCMC scheme as a separate reversible jump block. In each case, we wish to assume that all covariate models are equally likely a priori. We begin by assuming that all models of the same dimension are equally likely:

pγlG|qlG=cGqlG−1,G=C,B,I;l=1,…,4,

where *c* ^*C*^, *c* ^*B*^ and *c* ^*I*^ are the total numbers of available continuous, binary and interaction covariates, respectively. We then choose

$$q_{l}^{C}∼\text{Bin}\left(c^{C},0.5\right),\;\;\;q_{l}^{B}∼\text{Bin}\left(c^{B},0.5\right),\;\;\;p(q_{l}^{I})=\left\{\begin{array}{ll} \frac{1}{12} & \;q_{l}^{I}=0\\ \frac{4}{12} & \;q_{l}^{I}=1\\ \frac{6}{12} & \;q_{l}^{I}=2\\ \frac{1}{12} & \;q_{l}^{I}=3\\ 0 & \;q_{l}^{I}=4 \end{array}\right.,$$

where the latter prior excludes the possibility that $q_{l}^{I}=4$ as this would lead to an unidentifiable model and acknowledges that all four possible models with $q_{l}^{I}=3$ are essentially the same. Note that this specification renders equally probable a priori all distinct and identifiable models both within each covariate group *and* for the composite model defined by *γ*_*l*_ and *q*_*l*_.

### 3.4 Inference

#### 3.4.1 Hierarchical model

Let Ω denote the collection of all parameters in the population model for the *θ*_*ijkl*_s. For example, for the simple one‐level population model discussed in Section 3.2.1, $\Omega =\left(\varphi ,\beta ,\sigma_{\theta }^{2},\gamma ,q\right)$, where, here and throughout, a quantity not indexed by *i*, *j*, *k*, *l* or *m* represents the collection of *all* quantities sharing the same variable name, for example, *φ* = (*φ*_*l*_)_*l* = 1,…,4_. The joint posterior distribution under the hierarchical model is then given by

(4) p(Ω,θ,κ,λ∣y)∝p(y∣θ,κ,λ)p(κ)p(λ)p(θ∣Ω)p(Ω).

We make inference for the full hierarchical model (4) through a two‐stage approach 13, which is outlined as follows.

#### 3.4.2 Stage 1 analysis

In the first stage, we construct and estimate a posterior distribution for each profile independently, using the likelihood defined in Section 3.1.2, independent priors for the nuisance parameters given by (3) and independent, ‘flat’ priors for the kinetic parameters given by

(5) $$t_{\mathrm{ijk}}^{\max}∼\text{U}(5,500),\;\;{\text{MCR}}_{\mathrm{ijk}}∼\text{U}(0,0.25),\;\;a_{\mathrm{ijk}}∼\text{N}\left(0,100^{2}\right),\;\;b_{\mathrm{ijk}}∼\text{N}\left(0,100^{2}\right),$$

where the lower and upper bounds for *t* ^max^ and MCR represent the minimum and maximum physiologically plausible values, respectively. Using MCMC, we generate a sample of simulated values for the profile‐specific parameters from each profile‐specific posterior:

(6) $$p_{1}\left(\theta_{\mathrm{ijk}},\kappa_{\mathrm{ijk}},\lambda_{\mathrm{ijk}}|y_{\mathrm{ijk}}\right)\propto p\left(y_{\mathrm{ijk}}|\theta_{\mathrm{ijk}},\kappa_{\mathrm{ijk}},\lambda_{\mathrm{ijk}}\right)p\left(\kappa_{\mathrm{ijk}}\right)p\left(\lambda_{\mathrm{ijk}}\right)p_{1}\left(\theta_{\mathrm{ijk}}\right),$$

where *y*_*ijk*_ denotes the set of all measured concentrations for profile *i* *j* *k* and *p*_1_(*θ*_*ijk*_) is the ‘stage 1 prior’ for *θ*_*ijk*_, given by the product of terms in (5). We denote the samples by $\left\{\theta_{\mathrm{ijk}}^{\left(h\right)},\kappa_{\mathrm{ijk}}^{\left(h\right)},\lambda_{\mathrm{ijk}}^{\left(h\right)}\right\}$, *h* = 1,…,*H*_*ijk*_, and store them for use in stage 2, where they will be used to form proposal distributions for the profile‐specific parameters in the full hierarchical model.

#### 3.4.3 Stage 2 analysis

Stage 2 defines an MCMC scheme for updating all unknown parameters in the full hierarchical model. The parameters for each of the 12 covariate selection sub‐models, $\beta_{l}^{G}$, $\gamma_{l}^{G}$, $q_{l}^{G}$, *G* = *C*,*B*,*I*, *l* = 1,…,4, are jointly updated using reversible jump MCMC as described elsewhere 12. The remaining parameters in Ω are updated by standard means. Each of the ‘intercept’ parameters, *φ*_*l*_ and, where appropriate, *ψ*_*il*_, $\chi_{\mathrm{ijl}}^{V}$ and $\chi_{\mathrm{ikl}}^{M}$, *i* = 1,…,*N*, *j* = 1,…,*J*_*i*_, *k* = 1,…,*K*_*ij*_, *l* = 1,…,4, has a full conditional distribution that is available in closed form, and so a standard Gibbs step is appropriate. The variance components, $\sigma_{\theta_{l}},\sigma_{\psi_{l}},\sigma_{\chi_{l}^{V}},\sigma_{\chi_{l}^{M}}$, *l* = 1,…,4, can be updated by slice sampling 14, say, which is the default option in OpenBUGS for our model.

The profile‐specific parameters *θ*_*ijk*_, *κ*_*ijk*_ and *λ*_*ijk*_ are updated, jointly, as follows. From (4), their joint full conditional distribution is given by

(7) $$p\left(\theta_{\mathrm{ijk}},\kappa_{\mathrm{ijk}},\lambda_{\mathrm{ijk}}|\Omega ,y\right)\propto p\left(y_{\mathrm{ijk}}|\theta_{\mathrm{ijk}},\kappa_{\mathrm{ijk}},\lambda_{\mathrm{ijk}}\right)p\left(\kappa_{\mathrm{ijk}}\right)p\left(\lambda_{\mathrm{ijk}}\right)p\left(\theta_{\mathrm{ijk}}|\Omega \right).$$

We wish to make a Metropolis–Hastings update with this as the *target* distribution. A candidate update $(\theta_{\mathrm{ijk}},\kappa_{\mathrm{ijk}},\lambda_{\mathrm{ijk}})\to \left(\theta_{\mathrm{ijk}}^{⋆},\kappa_{\mathrm{ijk}}^{⋆},\lambda_{\mathrm{ijk}}^{⋆}\right)$ is drawn from the *proposal* distribution by choosing *s* uniformly from {1,…,*H*_*ijk*_} and setting $\left(\theta_{\mathrm{ijk}}^{⋆},\kappa_{\mathrm{ijk}}^{⋆},\lambda_{\mathrm{ijk}}^{⋆}\right)=\left(\theta_{\mathrm{ijk}}^{\left(s\right)},\kappa_{\mathrm{ijk}}^{\left(s\right)},\lambda_{\mathrm{ijk}}^{\left(s\right)}\right)$, where the right‐hand side is one of the samples stored in stage 1. From (6) and (7), the *target‐to‐proposal density ratio* is thus

$$R\left(\theta_{\mathrm{ijk}}\right)\propto \frac{p\left(\theta_{\mathrm{ijk}}|\Omega \right)}{p_{1}\left(\theta_{\mathrm{ijk}}\right)}.$$

Note the cancellation of likelihood terms and nuisance priors. This makes for rapid computation in stage 2, facilitating exploration of a wide range of population models for the profile‐specific parameters. That the ratio does not depend on *κ*_*ijk*_ or *λ*_*ijk*_ also means that these parameters need not actually be updated. The Metropolis–Hastings acceptance probability for the proposed update is min(1,*ρ*), where *ρ* is given by the target‐to‐proposal ratio at the proposed state divided by that at the current state:

$$\rho =\frac{p\left(\theta_{\mathrm{ijk}}^{⋆}|\Omega \right)}{p\left(\theta_{\mathrm{ijk}}|\Omega \right)}\times \frac{p_{1}\left(\theta_{\mathrm{ijk}}\right)}{p_{1}\left(\theta_{\mathrm{ijk}}^{⋆}\right)}.$$

If the stage 1 prior *p*_1_(.) is ‘flat’, as in our model, then the ratio on the right can be ignored as it is approximately equal to 1.

### 3.5 Model assessment via cross‐validation

The two‐stage method described earlier also expedites cross‐validation to assess the various models considered. In leave‐one‐out cross‐validation, a model is evaluated via predictions drawn from the model estimated with a single observation, or set of observations, excluded. Large disparities, measured by a discrepancy function, between these predictions and the excluded observations are indicative of model inadequacy. The procedure is repeated with each observation, or set of observations, omitted in turn. Such approaches have been widely discussed in the literature 15, 16.

Note that here we wish to assess the population model for the random effects *θ*_*ijk*_, as opposed to the fit of the pharmacokinetic model to the observed response data. A discrepancy function defined in terms of the response data is not appropriate when the focus is on the random effects, because agreement or otherwise of random effect estimates is unnecessarily masked by the observation error. Hence we require a discrepancy function that measures differences between ‘observed’ and ‘predicted’ random effects, denoted $\theta_{\mathrm{ijk}}^{\text{obs}}$ and $\theta_{\mathrm{ijk}}^{\text{pred}}$, respectively. Clearly, the random effects cannot be observed, but the profile‐specific posteriors defined by (6) can be used in lieu of observations 15. The predicted random effects are obtained from the ‘predictive prior’:

(8) $$p\left(\theta_{\mathrm{ijk}}^{\text{pred}}|y_{\setminus \mathrm{ijk}}\right)=\int p\left(\overset{\text{pred}}{\underset{\mathrm{ijk}}{\theta }}|\Omega \right)p\left(\Omega |y_{\setminus \mathrm{ijk}}\right)d\Omega ,$$

where *y*_∖*ijk*_ denotes all observations except those from individual *i* during and the following meal *k* of visit *j*. This must be estimated with observations from each profile excluded in turn, which can be prohibitively time‐consuming if the model is complex, as here. However, with the two‐stage methodology presented here, only the second stage (which is computationally quick) needs to be repeated each time. Hence, this is a potentially important alternative to importance sampling 17, which can be unstable, or approximation 15, 18.

Let $D\left(\theta_{\mathrm{ijk}}^{\text{obs}},\theta_{\mathrm{ijk}}^{\text{pred}}\right)=\theta_{\mathrm{ijk}}^{\text{pred}}-\theta_{\mathrm{ijk}}^{\text{obs}}$ be our discrepancy function. We define the Bayesian *p*‐value $P=\mathrm{Pr}\left(D\left(\overset{\text{obs}}{\underset{\mathrm{ijk}}{\theta }},\theta_{\mathrm{ijk}}^{\text{pred}}\right)\leq 0|y\right)$ and estimate it, for each profile, by independent sampling from the predictive prior (8) and the stage 1 posterior (6), obtaining *n* *p*‐values in total. Each *p*‐value should be uniformly distributed under the ‘true’ sampling model 15, suggesting that model assessment guided by quantile–quantile plots of *p*‐values is appropriate (note, however, that the *p*‐values are not independent).

## 4 Results

### 4.1 Stage 1 analysis

To draw samples from the independent, profile‐specific posterior distributions, we used the freely available BUGS software 2, 19. Specifically, we used the OpenBUGS implementation (www.openbugs.net), which allows regression functions to be specified in terms of differential equations as standard. Convergence of the generated Markov chains was assessed informally by visually examining chain‐history plots and formally by applying the Brooks–Gelman–Rubin diagnostic 20, 21 to the output from two Markov chains starting from widely differing initial values. We found that a burn‐in phase of 100000 iterations was easily sufficient. We performed a further 1000000 iterations following burn‐in for each profile, retaining every 100th value of each kinetic parameter (giving 10000 approximately independent samples).

**Figure 3:**
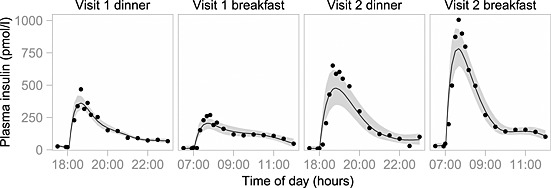
Four profiles from a typical individual: study 1, subject 1. The solid line is the (stage 1) posterior median model fit, and the shaded region is the 95% credible interval. The black circles (∙) are the observed insulin concentrations. Note that some of the variabilities between these plots are due to differences in the input (bolus sizes were 8, 5, 20 and 20 international units, respectively).

**Figure 4:**
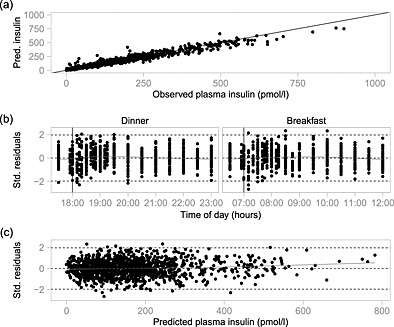
Model assessment following stage 1 analysis: (a) predicted (posterior median) versus observed insulin concentrations (∙) with identity line *y* = *x* (—); (b) posterior median standardised residuals versus time with mealtimes indicated by vertical lines (—); and (c) posterior median standardised residuals versus posterior median predicted concentration. The grey lines (gray—) are splines for the standardised residuals.

A total of 1302 plasma insulin concentrations were available for analysis. The model fits for a typical individual are shown in Figure 3. Our model was unable to fit six out of the 88 profiles available, and so these profiles were removed from our analysis. Figure 4(a) shows predicted (posterior median) versus observed insulin concentrations for all profiles fitted and indicates a good performance overall. To examine the model's performance in more detail, we also plot standardised residuals against time and against predicted concentration (Figure 4(b) and (c), respectively). There are no obvious trends, suggesting that the residual variance model is adequate and the residuals are generally within the expected range, although there is a hint of systematic bias about 10min after each mealtime.

### 4.2 Stage 2 analyses

**Figure 5:**
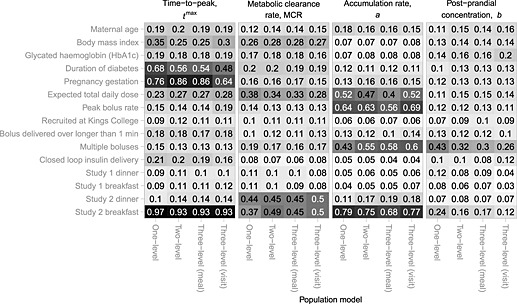
Marginal posterior inclusion probabilities for each of the 15 covariates considered under each of the four population models (including covariates) for each of the four kinetic parameters (*t* ^max^, MCR, *a* and *b*). The shading is proportional to the inclusion probability.

Figure 5 shows the posterior inclusion probabilities for each covariate being included in the linear predictor for each pharmacokinetic parameter, under each population model. We consider an inclusion probability greater than 0.5 to signify a notable association. The covariates identified as associated with each pharmacokinetic parameter were substantively consistent across the population models considered. All models strongly indicate that both *t* ^max^ and *a* differ after breakfast in study 2 compared with the other meals and studies. We also found evidence for association of *t* ^max^ with pregnancy gestation and diabetes duration and of *a* with peak bolus rate, expected total daily dose and delivery of multiple boluses. Evidence of association with MCR was less strong, although there was some evidence that MCR differs in study 2. No notable associations were observed for *b*.

**Figure 6:**
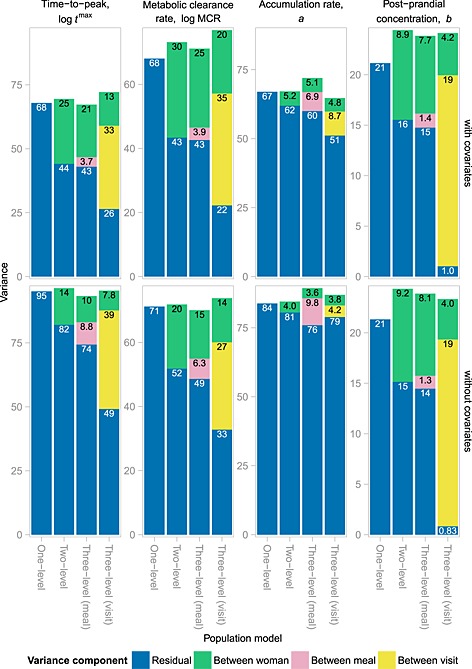
Decomposition of the variability in each pharmacokinetic (PK) parameter under each population model. Each panel represents the variance decomposition for a particular PK parameter for models either with (top row) or without (bottom row) covariates. Each coloured bar represents the proportion of total variance in the corresponding component for one of the four population models considered. Numbers shown within bars and on the *y*‐axes are variances multiplied by the following factors, for log*t* ^max^, logMCR, *a* and *b*, respectively: ×10^3^, ×10^3^, ×10^4^ and ×10^−2^.

Cross‐validation analyses demonstrated that all of the population models we consider perform well: quantile–quantile plots (not shown) of the distribution of *p*‐values under each model showed no obvious departures from uniformity. It was not possible to discern whether any specific model gave ‘more uniform’ plots than others. Differences amongst the models are more evident in the variance decomposition. These are shown in Figure 6. Two conclusions in common are apparent for *t* ^max^ and MCR when comparing the various models. First, the models including covariates exhibit notably less residual variability, as might be expected given the evidence of parameter–covariate association described earlier. Second, the residual variability is reduced in the two‐level and three‐level models compared with the one‐level model. The three‐level, meal‐specific model, however, offers little beyond the two‐level model in terms of explaining variability of the random effects: the residual variability is almost identical. In contrast, the three‐level, visit‐specific model substantially reduces the residual variability beyond that in the two‐level model. The two‐level and three‐level models are similarly an improvement for the post‐prandial concentration *b*, but there is little difference between the models with and without covariates. Conversely, woman‐specific effects are less apparent for the accumulation rate *a*, but the inclusion of covariates does make a notable difference to the residual variability.

Overall, the three‐level, visit‐specific model with covariates appears to offer the best explanation of the observed variation. Parameter estimates for this model are shown in Table 2. The most notable effect is the faster absorption after breakfast in study 2 as implied by the substantially decreased time‐to‐peak *t* ^max^ for these profiles. We also estimated that time‐to‐peak *t* ^max^ increases by 1.7% per week of gestation in pregnancy but decreases by 1.1% per year of diabetes.

**Table 2:** Insulin aspart pharmacokinetics in type 1 diabetes pregnancy.

	Aspart PK parameter			
Factor	Time‐to‐peak, *t* ^max^ (min)	Metabolic clearance rate, MCR (l/kg/min)	Accumulation rate, *a* (pmol/l/min)	Post‐prandial concentration, *b* (pmol/l)
Typical parameters for each				
study/mealtime combination				
Study 2 breakfast	**41**(**3.9**)	**0.028**(**0.0030**)	**0.056**(**0.044**)	33(12)
Study 2 dinner	57(4.7)	**0.022** **(** **0.0025** **)**	−0.047(0.033)	30(11)
Study 1 breakfast	55(3.8)	0.025(0.0021)	−0.029(0.027)	31(10)
Study 1 dinner	56(3.9)	0.025(0.0021)	−0.034(0.027)	31(10)
Effect sizes				
Peak bolus rate (U/h)	0.85(1.1)%	0.11(1.2)%	**0.012**(**0.0048**)	0.49(1.4)
Pregnancy gestation (week)	**1.7**(**0.73**)%	−0.33(0.87)%	−0.0039(0.0032)	0.47(1.4)
Multiple boluses	−11(14)%	17(15)%	**0.12**(**0.049**)	39(24)
Expected total dose (U/day)	0.37(0.36)%	−0.45(0.33)%	**−0.0027**(**0.0012**)	−0.19(0.62)
Duration of diabetes (year)	**−1.1**(**0.64**)%	0.66(0.76)%	0.0021(0.0024)	−0.12(1.2)

Top: posterior means (standard deviation (s.d.)) of each pharmacokinetic (PK) parameter for each study and mealtime combination for ‘typical’ covariate values, as defined by setting all continuous and binary covariates (except study–mealtime interactions) to zero. Bottom: posterior means (s.d.) of the effect sizes for covariates with a posterior inclusion probability >0.5 in at least one model – estimates are conditional on inclusion of the corresponding covariate. The effect sizes shown for *t* ^max^ and MCR are estimated *percentage* changes per unit change in the covariate; for *a* and *b*, we show the estimated *absolute* change. All notable effects are indicated in bold.

## 5 Discussion

We have presented a two‐stage method for simplifying the analysis of complex hierarchical data. This can be thought of as a type of particle filtering (sequential Monte Carlo sampling; 22, 23), where the resampling is carried out via Metropolis–Hastings. The method can be used whenever there is sufficient information in unit‐specific data that units can be analysed independently (in stage 1). When unit‐specific models are complex, as here, independent analyses may be a prerequisite for a full hierarchical analysis anyway, because the unit‐specific data sets may require individual attention, for example, because of convergence issues or because we wish to assess whether a given unit‐specific model can perform adequately over a wide range of units, say. However, our method is most likely to be useful when there is a range of ‘population’ models to consider for the unit‐specific parameters. This is because the likelihood is dealt with in stage 1 and need not be computed in stage 2. Hence, the second stage can be performed repeatedly at little computational cost. This has allowed us to explore parameter–covariate relationships between four unknown parameters and 13 covariates of three different types, under a range of hierarchical structures (with up to four levels), for a model defined in terms of differential equations. The two‐stage approach has also greatly facilitated cross‐validation analyses to assess model performance. We chose to use cross‐validation to explore models because we wished to focus our assessment of the models specifically on the choice of random effects model. Alternative approaches, such as the deviance information criterion (DIC) 24, would have undesirably focused on the pharmacokinetic model instead. It is our belief that the analyses presented herein would have been practically infeasible (or at least extremely cumbersome and time‐consuming) without a two‐stage approach.

We are mainly interested in the parameters *t* ^max^ and MCR, because *a* and *b* relate to the ‘residual’ insulin model, which is somewhat speculative (although it may aid in compensating for model misspecification). Our analyses indicate that *t* ^max^ is related to diabetes duration and gestational age in pregnant women using CSII. Time‐to‐peak increases by 1.7% per week of gestation and decreases by 1.1% per year of diabetes. In addition, there was a faster time‐to‐peak after breakfast in study 2, suggesting that moderately vigorous physical activity may counteract the gestational delay. The factors contributing to slower *t* ^max^ in late pregnancy are unknown. The impact of diabetes duration may be related to loss of residual C‐peptide activity 25. While these clinical/demographic factors are not easily modified, the impact of exercise, most likely related to enhanced tissue perfusion and temperature, is potentially modifiable and may be a useful tool for speeding up insulin absorption as pregnancy advances. In contrast with Gagnon‐Auger *et al.* 26, where substantially higher doses were used, our results do not indicate relationships between *t* ^max^ and prandial dose, total daily dose or maternal body mass index.

There were no highly probable relationships identified for *MCR*, although in some models, there was a suggestion of decreased clearance following study 2 dinner and increased clearance following study 2 breakfast. This latter effect could be due to increased blood flow following physical exercise. There were also no relationships identified for *b*, but several were apparent for the drainage rate (−*a*). The drainage rate increased as the expected total daily dose increased but decreased with peak bolus rate and when multiple boluses were used.

A limitation of our analysis is that the data apply only to aspart delivered by CSII and are not necessarily applicable to lispro (another rapid‐acting insulin analogue) or multiple daily injection therapy. However, as noted by Homko *et al.* 27, aspart and lispro have comparable pharmacokinetics, and CSII is increasingly recommended when glycaemic control targets are not achieved on multiple daily injections 28.

Although the two‐stage method does not require independence between the four kinetic parameters, we chose in Section 3.2 to make separate distributional assumptions for each parameter. This is to avoid being too informative about the sizes (and relative sizes) of the variance components in our model, which characterise both between‐patient and within‐patient variabilities. The obvious alternative would be to assume multivariate normality for the vector (log*t* ^max^, logMCR,*a*,*b*)′, with a covariance matrix free to assume any symmetric–positive–semidefinite (SPSD) form, allowing for any correlations that may exist between the different kinetic parameters. The inverse‐Wishart prior commonly chosen for such covariance matrices ensures the SPSD constraint automatically. However, it is more informative than might be expected and can have considerable influence on the posterior, particularly when, as here, there are multiple levels of variability and small sample sizes within levels (e.g. 29). Other approaches proposed in the literature (e.g. 30, 31) may be less informative, but we have chosen the relatively simple option of not allowing for correlations between kinetic parameters and assuming uniform priors for their variances. We feel that this approach also reduces the possibility of confounding between covariance parameters and the inclusion of covariate effects.
